# HIV testing experiences and their implications for patient engagement with HIV care and treatment on the eve of ‘test and treat’: findings from a multicountry qualitative study

**DOI:** 10.1136/sextrans-2016-052969

**Published:** 2017-07-23

**Authors:** Alison Wringe, Mosa Moshabela, Constance Nyamukapa, Dominic Bukenya, Ken Ondenge, William Ddaaki, Joyce Wamoyi, Janet Seeley, Kathryn Church, Basia Zaba, Victoria Hosegood, Oliver Bonnington, Morten Skovdal, Jenny Renju

**Affiliations:** 1 Department of Population Health, London School of Hygiene and Tropical Medicine, London, UK; 2 University of KwaZulu Natal, Durban, South Africa; 3 African Health Research Institute, KwaZulu-Natal, South Africa; 4 Biomedical Research and Training Institute, Harare, Zimbabwe; 5 Medical Research Council/Uganda Virus Research Institute Research Unit on AIDS, Entebbe, Uganda; 6 Kenya Medical Research Institute, Kisumu, Kenya; 7 Rakai Health Sciences Program, Kalisizo, Uganda; 8 National Institute for Medical Research, Mwanza, Tanzania; 9 Department of Global Health, London School of Hygiene and Tropical Medicine, London, UK; 10 Department of Social Statistics and Demography, University of Southampton, Southampton, UK; 11 University of Copenhagen, Copenhagen, Denmark; 12 Biomedical Research and Training Institute, Harare, Zimbabwe; 13 Malawi Epidemiology and Intervention Research Unit, Karonga, Malawi

**Keywords:** HIV testing, Africa, qualitative research, adherence, biopolitics, governmentality

## Abstract

**Objective:**

In view of expanding ‘test and treat’ initiatives, we sought to elicit how the experience of HIV testing influenced subsequent engagement in HIV care among people diagnosed with HIV.

**Methods:**

As part of a multisite qualitative study, we conducted in-depth interviews in Uganda, South Africa, Tanzania, Kenya, Malawi and Zimbabwe with 5–10 health workers and 28–59 people living with HIV, per country. Topic guides covered patient and provider experiences of HIV testing and treatment services. Themes were derived through deductive and inductive coding.

**Results:**

Various practices and techniques were employed by health workers to increase HIV testing uptake in line with national policies, some of which affected patients’ subsequent engagement with HIV services. Provider-initiated testing was generally appreciated, but rarely considered voluntary, with instances of coercion and testing without consent, which could lead to disengagement from care.

Conflicting rationalities for HIV testing between health workers and their clients caused tensions that undermined engagement in HIV care among people living with HIV. Although many health workers helped clients to accept their diagnosis and engage in care, some delivered static, morally charged messages regarding sexual behaviours and expectations of clinic use which discouraged future care seeking. Repeat testing was commonly reported, reflecting patients’ doubts over the accuracy of prior results and beliefs that antiretroviral therapy may cure HIV. Repeat testing provided an opportunity to develop familiarity with clinical procedures, address concerns about HIV services and build trust with health workers.

**Conclusion:**

The principles of consent and confidentiality that should underlie HIV testing and counselling practices may be modified or omitted by health workers to achieve perceived public health benefits and policy expectations. While such actions can increase HIV testing rates, they may also jeopardise efforts to connect people diagnosed with HIV to long-term care, and undermine the potential of test and treat interventions.

## Background

Despite expanding HIV testing opportunities, only an estimated 60% of people living with HIV (PLHIV) were aware of their HIV status in sub-Saharan Africa by 2015.^1^ Furthermore, among diagnosed PLHIV, poor linkage to HIV care and treatment, as well as suboptimal retention, are common challenges that threaten to undermine the viability of the universal test and treat (UTT) approach.^2 3^ Studies exploring the underlying reasons for poor linkage and retention in care have shown that psychosocial factors (including readiness for life-long care in the face of pervasive HIV stigma), as well as persistent social and structural factors undermine engagement with care.^4–7^ However, there is a dearth of research which considers how the experiences of PLHIV during their encounters with HIV testing services may influence their subsequent engagement with HIV care and treatment.

A growing body of work has shown how some of the principles that should underpin all forms of HIV testing, particularly consent and confidentiality,^8^ may be modified or omitted by healthcare workers (HCW) in African settings, with medical or public health imperatives sometimes cited to justify testing clients without their explicit consent or informing partners about a client’s HIV test results.^9–12^ In this paper, we expand on this body of work by exploring how the applications of these underlying principles of HIV testing can shape the experience of learning one’s HIV positive status and have consequences for patients’ subsequent engagement in HIV care.

Our analysis draws on Foucault’s notion of governmentality^13^ previously applied to other health service domains^14–16^ to consider the way in which patients are managed or governed through organised practices, rationalities and techniques within the clinical setting to ensure the fulfilment of government policies related to HIV testing. In line with the arguments laid out by Paparini *et al*, we also apply a broader ‘biopolitical’ lens to consider how the application of national HIV testing policies within health facilities may directly impact on biological aspects of the lives of PLHIV, namely their health and well-being which result from their subsequent engagement (or not) with HIV treatment.^17^ This biopolitical approach enables us to examine how the act of diagnosing HIV among the population, when viewed by governments or institutions as a scientific, political and biological problem,^18^ can lead to exertions of control over PLHIVs’ knowledge within the clinical setting. Examining these interactions, and the responses of PLHIV vis-à-vis their attitudes to HIV care, through the narratives of HCW and PLHIV, can provide a deeper understanding of how the HIV testing experience can influence subsequent engagement in HIV care.

## Methods

We use data from a multicountry qualitative study investigating bottlenecks in HIV care in seven health and demographic surveillance sites (HDSS) in Eastern and Southern Africa: Kisesa (Tanzania), Kyamulibwa and Rakai (Uganda), Kisumu (Kenya), Karonga (Malawi), uMkhanyakude (South Africa) and Manicaland (Zimbabwe). All sites are located in rural areas with generalised epidemics, where government-run HIV treatment services have been available since 2004–2005.^19^

In each setting, participants were purposively sampled either from HIV clinics or associated services, or from sampling frames that were constructed using HDSS databases and clinic records. In total, 264 PLHIV (93 men, 127 women) with a range of HIV care and treatment histories participated (table 1). HCW were purposively sampled to ensure a range of perspectives on HIV service provision from different cadres.

**Table 1:** Description of study participants by site

Country	HDSS	HCW	Persons living with HIV		
			Never on ART	On ART	LTFU*
Uganda	Rakai	6	15	15	6
Uganda	Kyamulibwa	5	8	16	4
Kenya	Kisumu	8	10	15	6
Tanzania	Kisesa	7	13	14	4
Malawi	Karonga	5	9	27	4
Zimbabwe	Manicaland	4	16	35	8
South Africa	uMkhanyakude	19	16	17	6
	Total	54	87	139	38

*Loss to follow-up from an HIV clinic for >90 days.

ART, antiretroviral therapy; HCW, healthcare worker; HDSS, health and demographic surveillance sites; LTFU, loss to follow-up.

Participants were recruited by fieldworkers or health facility staff, and in-depth interviews (IDI) lasting 60–90 min were conducted by fieldworkers in the local language. IDIs were undertaken in private, either at the health facility or the participants’ homes according to interviewees’ preferences. Topic guides covered patients’ and HCW experiences with the receipt or provision of HIV services. Written informed consent was obtained. Participants were provided with a small compensation for their time or travel expenses.

Interviews were audio-recorded, transcribed and translated into English in all sites except Kyamulibwa where detailed case summaries were prepared after each interview. Data were coded by the study coordinator in each setting, with Nvivo or manually, using a framework analysis approach to identify emerging patterns and relationships between the codes.^20^ The data were then centralised by the first author, and an inductive coding approach was used to identify emerging themes which were discussed at regular intervals with the study coordinators and refined as necessary.

Ethical approval was obtained from the relevant authorities in each country and the London School of Hygiene and Tropical Medicine, UK.

Further details on the study methods can be accessed from the online Supplementary Methods found at http://dx.doi.org/10.1136/sextrans-2017-053172.

## Results

### Rationalities and techniques associated with the HIV testing process and their consequences for care engagement

HIV testing through provider-initiated models was generally appreciated by PLHIV but was not always perceived to be a choice by counsellors or those undergoing testing. This was frequently apparent in the accounts of both HCW and pregnant women who described HIV testing during antenatal care:

*Int: Was there any motivation to make the decision to… to go for (HIV) testing?*

*Part: Of course, there wasn’t any decision. Just a nurse told me: ‘Go over there for testing’. (Female, pre-ART, East Africa)*

For both clients and HCW, the rationale that justified this apparent lack of an opportunity for opting-out from testing was the fact that it was perceived to be ‘an instruction of the hospitals’, ‘mandatory’, ‘compulsory’ or ‘government policy’. There were also alternative explanations for the lack of opportunities to opt-out, with one woman suggesting that counsellors were required to test a certain number of clients to be paid:

*At the end of the day they should have a number of people who tested positive so that they can have their salary at the end of the month. That is why I was also the victim of being someone who tested positive. (Female, lost to follow-up, Southern Africa)*

Although explicit consent to test for HIV was usually sought, HCW adopted various techniques to convince reticent clients to learn their status. In some cases, they repeated counselling until clients finally agreed to undergo a test, either being brought round to their way of thinking or because they felt ‘forced’. Instances of coercion were also reported from most settings, whereby threats of service withdrawal for themselves or their children would be used to extract agreement to test:

*If they decline HIV testing, we tell them that they cannot get treatment from our facility. For example, pregnant mothers receive pills for prevention of anaemia… she may not use them appropriately if she declines HIV testing. Therefore, we tell them that if they refuse HIV testing, we cannot give them any health services until they have tested for HIV. (HCW, Eastern Africa)*

Clients responded in different ways to these techniques, with some consenting to test just to secure the services or drugs that they had initially been seeking:

*I went to the clinic because I was… having headache, then the Nurses said I must go and do an HIV test. The Nurses said they will not give me treatment for headache until I go for testing. I didn’t plan to go to test. I tested because I wanted the Nurses to give me the treatment for the headache. (Female, lost to follow-up, Southern Africa)*

Many, having learnt their status, subsequently engaged in HIV care:

*She found that I had a sickness (HIV) which deserved that treatment, so I had no alternative… I accepted to take the drugs, because I had no option. (Female, on ART, Eastern Africa)*

While some PLHIV appeared ambivalent about this outcome, others explicitly appreciated the concerted efforts that HCW had gone to get them to learn their positive HIV status, so that they could initiate antiretroviral therapy (ART), illustrated by one pregnant woman:

*I think they just do well testing for HIV virus when you are pregnant. You know, if you go to clinic, they’d just examine the pregnancy; they wouldn’t carry out a test for HIV virus. So, you’d just continue breast-feeding a baby; eventually the baby acquires the infections. I think they just do well testing for HIV virus when you are pregnant. (Female, On ART, Eastern Africa)*

However, PLHIVs responses to ‘submitting’ to HIV testing also included subsequent disengagement from care, throwing away ART or not returning to the clinic. As one woman explained:

*When they told me that I am HIV positive… I believed the results I had no choice. I wouldn’t argue with them, whatever they say I must do I will do it. I did all what they wanted me to do like taking bloods, attending sessions, and initiated by Nurse, but at the end… what they want me to do [take ART]… I will not do it!! (Female, lost to follow-up, Southern Africa)*

The practice of mandatory HIV testing in the context of antenatal care also led some women to seek care from traditional birth attendants or private facilities instead.

Other techniques that were employed to ensure that HIV testing was undertaken included colluding with other family members. A woman from Southern Africa recounted how she was tricked by her two sisters into bringing her child to the health centre where he was then tested for HIV without her consent:

*We… went there and my other sister told me that we should come out of the car (laughter) so that we should buy something… Since that [health worker] was told what the car looked like and its number plate, she just came to test the child. When I came back, they showed me the results of my child that he is positive! (Female, on ART, Southern Africa)*

In other cases, confidentiality was breached, with HCWs informing family members of the HIV test results without the explicit consent of the client. In most instances, this occurred when the person being tested was very ill and needed considerable care from a family member, particularly to access ART, thereby potentially enabling engagement in care. In one example in Southern Africa, the brother of a recently deceased PLHIV recalled how a HCW had told him his brother’s positive HIV status in front of him, without the brother’s permission, when he was very ill, to ensure that he could obtain the drugs from the clinic on his behalf.

While disclosure of a patient’s HIV status under such circumstances was seen to be necessary to save the patient’s life, it could still contribute to a perception that HCW were not always appropriately discreet about the HIV status of their clients. Even the possibility or rumours of HCW disclosure of HIV test results reportedly put people off learning their HIV status and engaging in HIV care in some of the study settings:

*Why do you think they were hiding?*

*R: I can’t know the reason…… Sometimes it was because they knew that you people […] don’t maintain confidentiality… so if we can meet you can tell other people about my status, you see. So this was one of the reasons that made people to run away… When they see people who are their friends or classmates working there, they were thinking that if I can explain my problems to him, he can in turn tell other people. So… I can’t accept to test… (Male, pre-ART, Southern Africa)*

### Rationalities underlying counselling practices and patient responses

There was often a degree of disconnect between the information sought by PLHIV during the HIV testing process and the counselling messages provided at the clinics, and this could influence attitudes regarding future care-seeking. The rationale driving many PLHIV to seek HIV testing was the desire to ‘check’ or ‘know’ their status, which contrasted with the perspective of some HCWs that the session was an opportunity to deliver morally laden messages regarding sexual or social behaviours:

*They’d tell you: ‘Do you want to live?’ They started by asking me: ‘Do you take alcohol?’ And I said: ‘I don’t take alcohol …I take soda only.’ ‘Ahaa, quit alcohol! And then quit women if you want to live for many years… quit women!’ (Male, pre-ART, Eastern Africa)*

*They also advised us that …we shouldn’t do sexual intercourse every day, but maybe once in a week. (Male, on ART, Southern Africa)*

Some HCW reported that this advice slowed down disease progression with one claiming that taking drugs, drinking alcohol or having unprotected sex could result in rapid CD4 count decline, particularly among youth. Patients responded in different ways to such advice, sometimes seeming to act on it, but where it seemed at odds with the realities of their lives, they often disregarded it.

*The health care providers give them education, but they listen and act as if they are going to do as they learnt, but they don’t follow the procedure like using the condom. (HCW, Southern Africa)*

However, there were also numerous instances in which counselling was reported to give hope to PLHIV, and to help them come to terms with their diagnosis. Indeed, it was often key in giving patients morale and desire to continue their HIV care-seeking journey:

*Most fell sick and get to a point where they are ashamed to come to the clinic to be tested, so for us we came to this clinic and we were taught not to be ashamed of our status. They told us that getting help and following instructions will make us survive, so we made a choice to take the medication and we are still taking it and I can see that there is a change from what I used to be back then and now. (Woman, On ART, Southern Africa)*

Furthermore, some participants responded by repeatedly undergoing HIV testing and counselling. Even once diagnosed, repeat testing and counselling was often part of the acceptance process or a means to check the accuracy of results or whether they were still infected after initiating ART and could encourage patients to continue with HIV care. Repeat testing also represented an opportunity to develop familiarity with clinics and build mutual trust with HCW which could further support engagement in care:

*I did not first accept [the results] because I was feeling healthy. After… 2 years, then my counsellor convinced me to accept them. He told me that ‘Sunday, let us test again’. I had many counsellors; Mrs X, Mr Y…and others but those two played a big role. (Male, on ART, Eastern Africa)*

## Discussion

We found that the circumstances under which PLHIV learnt their HIV status, notably if they were physically ill or pregnant, could influence the ways in which HCWs applied the principles of consent and confidentiality and modified the content of the counselling sessions. The resulting experience of being tested and counselled could promote or undermine subsequent care-seeking trajectories of PLHIV in a myriad of ways, highlighting the potential influence and importance of HIV testing services beyond merely increasing diagnosis rates, particularly as we enter the UTT era.

Foucault’s concept of governmentality posits that techniques, rationalities, institutions and knowledge can empower political programmes or policy implementation,^21^ while his notion of biopolitics, aids in considering the consequences of HIV testing policy implementation on patients’ engagement in HIV care and their associated health outcomes. In our study settings, as elsewhere, some of these techniques included insistence and persistence when obtaining consent or presenting testing as obligatory rather than an informed choice or at worst, coercing clients to test by threatening to withdraw future services for themselves or their children.^22^ As observed by others, we found that lapses of confidentiality, or failure to obtain consent, were generally imbued in a public health rationality, notably through discourses of protecting the health of an unborn or sick child, or trying to ensure that critically ill patients were given life-saving drugs by their relatives.^9 10 23^ Furthermore, as part of the governmentality process within HIV clinics, some HCW may sense an authority to apply such practices in the name of government policy implementation, legitimising the superiority of their knowledge and the morality of their decision making. Ironically, while coercive practices may result in increased HIV testing rates, thereby fulfilling national targets and aligning with policies, our findings suggest that they can be counter-productive by eroding trust in HIV services and undermining therapeutic alliances with HCW that could enhance subsequent adherence to treatment. The many benefits of ending HIV ‘exceptionalism’ and adopting widespread, routine HIV testing^24^ may being undermined by patients’ resistance to using HIV services if they perceive their choices to have been removed entirely.

Our findings align with other studies showing that the principles of consent and confidentiality are not always respected, with the omission of an opt-out clause in provider-initiated testing emerging as a widespread practice in many settings.^11 12 23–26^ However, few studies have considered patients’ responses to such practices,^27^ which we found to include resistance and disengagement from care. While it has been suggested that routine testing does not deter clients from accessing health services,^28^ our findings suggest that a failure to adhere to the principles of consent and confidentiality may have consequences for patients’ trust in, and future interactions with, HIV services, which are critical for effective, lifelong HIV care.

Our results also suggest that information provided during counselling sessions should be better aligned with clients’ rationales for undergoing an HIV test, and be tailored to their expressed concerns about learning their HIV status and seeking HIV care. Cawley *et al* have previously shown that counselling messages with directive and moralistic undertones that focus on the frequency of sexual encounters or insist on condom use without addressing its associated challenges are unlikely to engage clients in behaviour change to reduce HIV risks.^29^ The rationale for counsellors’ focus on behaviour change messages often stems from their perceived responsibility to minimise HIV spread by ‘educating’ PLHIV to reduce promiscuous behaviours.^29^ Our findings suggest that such messaging can also undermine fruitful discussions about PLHIVs’ future care-seeking concerns and represent a missed opportunity to engage patients in long-term care.

We also found that patient-initiated testing and re-testing could represent an enactment of agency in the context of uncertain HIV exposures and risks. Our findings align with Horter *et al*’s observations that the process of retesting, even for diagnosed persons, can be important in removing doubts over testing accuracy and promoting acceptance of an HIV diagnosis and subsequent engagement in care.^5^ Repeat counselling should thus be encouraged for PLHIV who initially struggle to accept their diagnosis to build up trust with HCW, address concerns about HIV and facilitate their subsequent engagement with HIV services.

Various limitations need to be considered when interpreting these findings including social desirability bias in participants’ accounts, despite extensive training of fieldworkers. The study’s strengths include our ability to recruit PLHIV who had never linked to care following a diagnosis, or who had defaulted, using the HDSS records as a sampling frame. Our ability to compare issues across several settings also demonstrated similarities in the governmentality of HIV testing programmes, including the practices and techniques used to secure high rates of testing uptake across different contexts.

In conclusion, we found that the principles that should underlie HIV testing and counselling practices may be modified or omitted by HCW to achieve perceived health benefits for PLHIV (or their entourage) or to achieve policy expectations. While such actions arguably save lives, they may also jeopardise efforts to connect many diagnosed PLHIV to the long-term HIV care necessary for elimination of AIDS by 2030.

**Key messages**

- Various practices and strategies are employed by health workers to increase HIV diagnosis rates and meet national policy expectations, some of which may undermine patients’ subsequent engagement with care.
- Rationalities for HIV testing among people living with HIV may not align with those of health workers. This discord can lead to missed opportunities to engage diagnosed persons in HIV care.
- Repeat testing among diagnosed patients often reflects doubts about test accuracy and hopes for a cure. Counselling should focus on (re-)engaging these patients in care.
- ‘Test and treat’ initiatives require long-term HIV care engagement which may be jeopardised if testing services omit the principles of consent, confidentiality and tailored counselling.
